# Automatic retinal nerve fiber bundle tracing based on large field of view polarization sensitive OCT data

**DOI:** 10.1364/BOE.443958

**Published:** 2021-12-03

**Authors:** Florian Schwarzhans, Sylvia Desissaire, Stefan Steiner, Michael Pircher, Christoph K. Hitzenberger, Hemma Resch, Clemens Vass, Georg Fischer

**Affiliations:** 1 Department of Clinical Pharmacology, Medical University Vienna, Vienna, 1090, Austria; 2 Center for Medical Statistics, Informatics and Intelligent Systems, Medical University Vienna, Vienna, 1090, Austria; 3 Center for Medical Physics and Biomedical Engineering, Medical University Vienna, Vienna, 1090, Austria; 4 Department of Ophthalmology and Optometry, Medical University Vienna, Vienna, 1090, Austria

## Abstract

A technique to accurately estimate trajectories of retinal nerve fiber bundles (RNFB) in a large field of view (FOV) image covering 45° is described. The method utilizes stitched projections of polarization-sensitive optical coherence tomography (PS-OCT) data, as well as a mathematical model of average RNFB trajectories as prior. The fully automatic process was applied to data recorded in healthy subjects and glaucoma patients and automatically detected individual RNFB trajectories are compared to manual traces.

## 1. Introduction

Glaucoma is a chronic optic neuropathy that severely damages the optic nerve head (ONH), causing a loss of the retinal nerve fiber layer (RNFL) and retinal ganglion cell (RGC) layer which consequently leads to visual field (VF) impairment [1–4]. The RNFL is composed by the RGC axons targeting the ONH where they leave the eye to travel towards the brain. The detection of RNFL damage by determining its thickness profile in comparison with normative values is one of the main sources in glaucoma diagnosis [5]. The measurement of RNFL thickness around the ONH is performed using imaging techniques such as optical coherence tomography (OCT) [6–11]. However, RNFL thickness varies considerably among healthy population, depending - among others - on age, sex, ethnicity and refractive error, as well as the location of the major retinal vessels [12–19]. This interindividual variation of RNFL thickness is a major obstacle for early and accurate glaucoma diagnosis.

When measured around the ONH, the detection of sectorial damage of the RNFL provides information on RGC loss in a retinal sector along the trajectory of the affected retinal nerve fiber bundle (RNFB). There may be substantial RGC loss before a VF defect is detected [3,20]. Most knowledge about the trajectories of the RNFBs is derived from manual tracing in fundus photographs of the nerve fibers, which poses several problems. First, manual measurement is not suitable for diagnostic routine due to the amount of time necessary. It was thus mainly used for generating mathematical models of average RNFB trajectories. Secondly the reproducibility of manual RNFB tracing is limited and consequently manual RNFB tracing is not suitable for generating maps with high spatial resolution as this severely increases the overall grading time.

Polarization sensitive OCT (PS-OCT), an extension of OCT, has previously been reported for the investigation of RNFB trajectories [21]. PS-OCT allows to retrieve additional contrast images over a 3D volume [22]. The polarization state of the light may be affected by different light-tissue interactions, such as depolarization and birefringence. In the retina, fiber based tissues, such as the RNFL and the Henle’s fiber layer, are known to be birefringent [23]. The axons of the RGCs are composed, among other intracellular structures, of microtubules (MTs). These MTs, oriented in parallel within the nerve fibers, are the main contribution to the RNFL form birefringence [24,25]. In the case of glaucoma, the RNFL birefringence may vary as a consequence of alteration of the MTs organization or a reduction of their number, as investigated in primate model of glaucoma [26].

Assessing RNFB birefringence is therefore of great interest for early disease diagnostic and monitoring. In order to evaluate thickness, retardation, and birefringence information along individual RNFBs, which may allow for a more accurate localization of glaucoma damage, as well as a more direct comparison between structure and function of the tissue, their orientation and trajectory need to be retrieved.

In reality early detection of glaucoma is similarly impeded by the interindividual variability of RNFL bundle trajectories and their measurement. Thus any method to reduce variability of the RNFL bundle tracing will improve the diagnostic performance.

In this work we propose a method of fully automatically reconstructing individual retinal nerve fiber bundle traces from PS-OCT based RNFL measurements in healthy subjects and glaucoma patients, as well as quantifying the reproducibility of said traces and comparing it to manual tracing.

## 2. Methods

### 2.1 Instrumentation set-up

The imaging system used was a custom-built prototype PS-OCT operating at 860nm with an A-scan rate of 70kHz and a FOV of 28° × 21° (1024 A-Scans × 250 B-Scans). An integrated line scanning laser ophthalmoscope (LSLO), operating at 790nm with a refresh rate of 60Hz, is used for tracking purposes [27,28]. The axial and lateral resolutions of the system are 4µm (in tissue) and 17µm ( $\frac{1}{e^{2}}$ intensity full width), respectively [29]. The lateral pixel spacing (assuming that 1° scanning angle corresponds to 300µm on the retina) is 8.2µm (x) and 25.2µm (y), the axial pixel spacing is 1.49µm in tissue. The system is controlled via a custom built LabView software that allows arbitrary positioning and movement of the OCT scanning beam along X and Y axis [30]. Additionally large FOV fundus photos (45° × 45°) were acquired using a separate Nonmyd WX-3D fundus camera (KowaCompany, Japan).

### 2.2 Data preparation

Volumetric PS-OCT raster scans each covering a FOV of 28° x 21° at 7 different retinal areas are recorded, with 3 consecutive acquisitions being recorded per area separately to increase the signal to noise ratio (SNR). These volumes are preprocessed, flattened and placed in the large FOV area defined by the fundus photography using the methods presented in [28]. The next step is to perform a retinal layer segmentation as it will be needed for subsequent steps. Particularly we are interested in the inner limiting membrane (ILM), the RNFL/ganglion cell layer (GCL) boundary, as well as the outer plexiform layer (OPL) / outer nuclear layer (ONL) boundary. Detecting retinal layers can be seen as an optimization problem which we tackle using a graph-based shortest path solution on a per B-Scan basis, similar to the implementation described in [28,31,32].

### 2.3 Subject selection

123 healthy subjects and 66 early glaucoma patients (mean deviation of visual field better than -6.0 dB) were acquired as part of an ongoing larger study. A subset containing 7 healthy subjects, as well as an additional 5 healthy subjects and 6 glaucoma patients that have undergone 3 scanning sessions is used for evaluating the methods proposed in this paper. These methods will later be used for evaluation of the entire data set. The study was approved by the university’s ethics committee and is in agreement with the tenets of the Declaration of Helsinki.

### 2.4 Estimation of nerve fiber orientation

With the data prepared the orientation of nerve fibers can be derived. There are various methods available for estimating nerve fiber orientation - each with unique benefits and drawbacks. Since these methods tend to perform better in some areas and worse in others we will be using multiple methods instead of only one. The ones we want to focus on are the mathematical model as developed by Jansonius et. al. [33,34], a polarization data based approach as proposed by Sugita et. al. [21] and a method considering pure intensity variations of the RNFL [35,36]. The orientation maps derived from those methods shall be denoted as $\phi_{M}$ , $\phi_{P}$ and $\phi_{F}$ for mathematical model, polarization data and fingerprint orientation estimation method, respectively. A detailed explanation follows below.

#### 2.4.1 Mathematical model

**Fig. 1.**
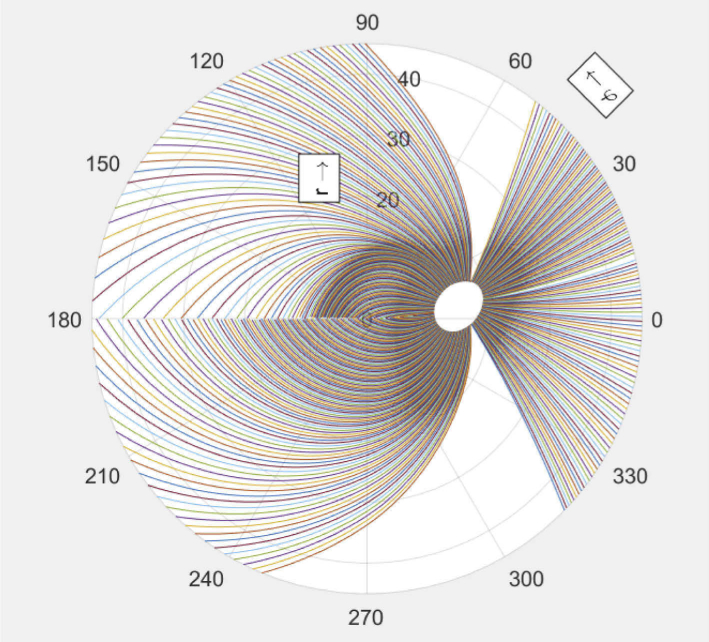
Polar plot of the RNFB trajectories as given by the mathematical model described in [33,34]. Fovea is located at position (0,0) in the center. The ONH is the source of all traces 15° to the right.

In [33] and [34] a mathematical model of the average trajectory of retinal nerve fibers is introduced by Jansonius et. al.. The parameters for that model were estimated based on manually assessed traces of nerve fiber bundles on fundus photos taken from 27 healthy subjects. A total of 1660 such traces (including 16816 sampling points) were used to estimate the model parameters. The model describes the trajectory of individual nerve fiber bundles using a second order polynomial in the form of

(1) $$\varphi (\varphi_{0},r)=\varphi_{0}+b(\varphi_{0})\times {\left(r-r_{0}\right)}^{c(\varphi_{0})}$$

with $\varphi_{0}=\varphi (r=r_{0})$ being the angular position of the trajectory at its starting point at a circle with radius $r_{0}$ around the center of the ONH. The model further defines the constants to be

(2) $$c=1.9+1.4\times \tanh\left(\frac{\varphi_{0}-121}{14}\right)$$

for the superior hemifield and

(3) $$c=1.0+0.5\times \tanh\left(\frac{-\varphi_{0}-90}{25}\right)$$

for the inferior hemifield, as well as

(4) $$\ln\left(b\right)=-1.9+3.9\times \tanh\left(-\frac{\varphi_{0}-121}{14}\right)$$

for the superior hemifield and

(5) $$\ln\left(-b\right)=0.7+1.5\times \tanh\left(-\frac{-\varphi_{0}-90}{25}\right)$$

for the inferior hemifield. Though these values are not individualized they give a very good overview of the rough trajectory of the retinal nerve fiber bundles. Using these parameters we have reimplemented the model (Fig. 1). The next step is to fit the given model to our data. Prior to that, however, the polar coordinate system is modified such that the position of the fovea is located at (0,0), as explained in detail in appendix A of [33].

**Fig. 2.**
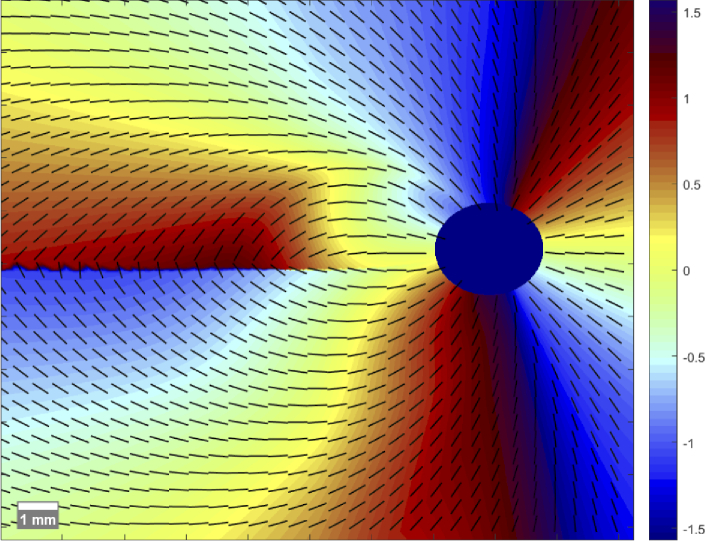
Interpolated orientation map derived from mathematical model (colormap in radian). Black bars drawn in regular intervals to further indicate the orientation vector.

For this we first create traces in resolution of 1 tracing per degree around the optic nerve head (see Fig. 1) and then interpolating these results on a grid with the dimensions of our large field of view projections. In order to fit the model, where the fovea is located exactly in the center of the image, the fovea position is adjusted by translation for each individual subject. The center of the ONH is then fitted by rotation and scaling (keeping the fovea position fixed), completing our orientation map of the mathematical model $\phi_{M}$ . Keep in mind that when rotating an orientation map ϕ by α degree, we have to compensate by also adding the value of α to the orientation values contained in ϕ . The fitted orientation map can be seen in Fig. 2 for a representative case.

#### 2.4.2 Polarization data

As previously mentioned, the RNFL is known to be birefringent whereas the subsequent layers, from the GCL to the OPL, do not affect the polarization state of the light. The polarization parameters, optic axis orientation θ and phase retardation δ , within the RNFL can thus be retrieved [37] by analyzing the OCT signal obtained from these polarization maintaining layers, after compensation of the corneal birefringence [23]. Near neighboring RNFBs being mainly oriented parallel to each other on a given position (x,y), the axis orientation is expected to be constant in depth. For a given retinal 3D volume, the RNFL retardation and axis orientation maps can be retrieved using Stokes vectors averaging with re-normalization, as described in [21]. After segmentation of the RNFL/GCL boundary (at depth locations $B_{NG}(x,y)$ ) and the OPL/ONL boundary (at depth locations $B_{OO}(x,y)$ ), the averaged normalized Stokes vectors ( $\left\langle \tilde{Q}\right\rangle$ , $\left\langle \tilde{U}\right\rangle$ , $\left\langle \tilde{V}\right\rangle$ ) are obtained as below:

(6) $$\left[\begin{array}{c} \left\langle \tilde{Q}(x,y)\right\rangle \\ \left\langle \tilde{U}(x,y)\right\rangle \\ \left\langle \tilde{V}(x,y)\right\rangle \end{array}\right]=\frac{1}{B_{OO}(x,y)-B_{NG}(x,y)}\times \sum_{i=B_{NG}(x,y)}^{B_{OO}(x,y)}\left[\begin{array}{c} \cos\left(2\delta (x,y,i)\right)\\ \sin\left(2\delta (x,y,i)\right)\times \cos\left(\pi -2\theta (x,y,i)\right)\\ \sin\left(2\delta (x,y,i)\right)\times \sin\left(\pi -2\theta (x,y,i)\right)) \end{array}\right].$$

The RNFL $\delta_{P}$ and $\phi_{P}$ components can then be obtained from:

(7) $$\left[\begin{array}{c} \delta_{P}(x,y)\\ \phi_{P}(x,y) \end{array}\right]=\frac{1}{2}\times \left[\begin{array}{c} \arccos\left({\left\langle \tilde{Q}(x,y)\right\rangle }_{R}\right)\\ \arctan(-\frac{{\left\langle \tilde{V}(x,y)\right\rangle }_{R}}{{\left\langle \tilde{U}(x,y)\right\rangle }_{R}}) \end{array}\right],$$

with $\left({\left\langle \tilde{Q}\right\rangle }_{R},{\left\langle \tilde{U}\right\rangle }_{R},{\left\langle \tilde{V}\right\rangle }_{R}\right)$ the re-normalized Stokes vectors defined as below:

(8) $$\left[\begin{array}{c} {\left\langle \tilde{Q}(x,y)\right\rangle }_{R}\\ {\left\langle \tilde{U}(x,y)\right\rangle }_{R}\\ {\left\langle \tilde{V}(x,y)\right\rangle }_{R} \end{array}\right]=\frac{1}{nrm(x,y)}\times \left[\begin{array}{c} \left\langle \tilde{Q}(x,y)\right\rangle \\ \left\langle \tilde{U}(x,y)\right\rangle \\ \left\langle \tilde{V}(x,y)\right\rangle \end{array}\right]$$

and

(9) $$nrm(x,y)=\sqrt{{\left\langle \tilde{Q}(x,y)\right\rangle }^{2}+{\left\langle \tilde{U}(x,y)\right\rangle }^{2}+{\left\langle \tilde{V}(x,y)\right\rangle }^{2}}.$$

**Fig. 3.**
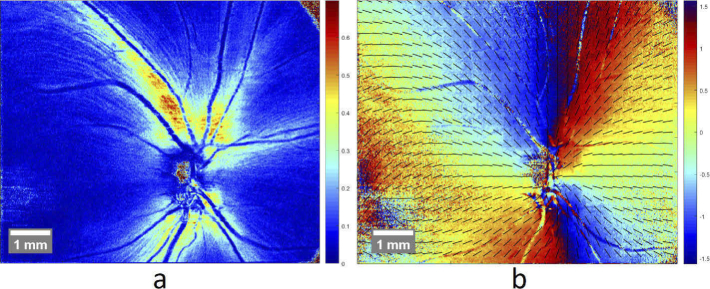
Averaged polarization data of the RNFL of one 3D volume scan area, showing the retardation (a) and axis orientation (b) of a healthy subject.

The resulting RNFL retardation and axis orientation maps of a 3D volume scan area from a healthy subject, including the ONH are given in Fig. 3. As the axis orientation is a relative value, derived from the difference between the two OCT channels [37], further compensation of the variable angle offset is required (see section 2.5).

#### 2.4.3 Nerve fiber layer intensity variation

**Fig. 4.**
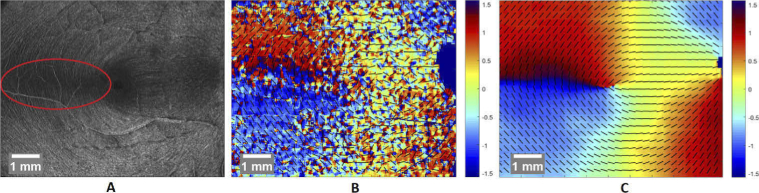
Projection of the RNFL, averaged near the RNFL/GCL boundary. Location of the raphe marked with red ellipse. Some RNFBs are clearly visible (A). Noisy orientation map derived via fingerprint orientation estimation techniques. Black bars drawn in to visualize direction (B). Further averaged and compensated orientation map (C).

Considering the retinal nerve fiber layer is made up of the nerve fiber bundles there are two ways to use the intensity channel of the volumetric raster scan OCT data to obtain information about the position/path of those bundles. One method would be to use retinal nerve fiber layer thickness data and checking the thickness variation as done in [21]. This is, however, highly dependent on the quality of the retinal layer segmentation and prone to failure in noisier data (as it often occurs in older and glaucoma subjects) and thus not our method of choice. Another way is to directly check the intensity value of the RNFL near the boundary to the ganglion cell layer (GCL). Since the RNFB have a higher reflectivity in the OCT scan they can be assessible by averaging the intensity near that boundary. Given an intensity OCT volume V(x,y,z) , a segmentation of the RNFL/GCL boundary $B_{NG}(x,y)$ and a segmentation of the ILM $B_{ILM}(x,y)$ - both describing the segmented layers by z-positions for all (x,y)-positions within the given volume V - we are able to create a nerve fiber projection image $NF_{MaxLen}(x,y)$ using

(10) $$NF_{MaxLen}(x,y)=\frac{1}{BE_{MaxLen}(x,y)}\times \sum_{i=1}^{BE_{MaxLen}(x,y)}V(x,y,B_{NG}(x,y)-i),$$

with (x,y) indicating the lateral positions of V and $BE_{MaxLen}(x,y)$ indicating the extent of the band at (x,y) -position in the z-dimension over which to average. To ensure that all these extents do not exceed the depth of the RNFL (to avoid including unwanted areas to $NF_{MaxLen}(x,y)$ ), $BE_{MaxLen}(x,y)$ is limited in size by MaxLen in $BE_{MaxLen}(x,y)$ as follows:

(11) $$BE_{MaxLen}(x,y)=min\left(B_{NG}\left(x,y\right)-B_{ILM}\left(x,y\right),MaxLen\right),$$

with MaxLen being our target band height which for our data delivered good contrast for a value of 15. As can be seen in Fig. 4(A) this projection image clearly shows the pathway of the RNFBs which is best seen close to the Raphe. The paths of RNFBs are for the most part smooth and (within a small window) fairly parallel and show in the image as a set of ridges and grooves. Fingerprint analysis is a widely studied field that deals with images that have very similar characteristics to our RNFB projection images, since there are ridges and grooves too, that are mostly oriented similarly within a small window. Since there are already several proposed algorithms for extracting local orientation data out of fingerprint images [38–43] we opted to use the underlying methods presented there, since they should be applicable to our image data with minimal modification. Our process for orientation estimation consists of the following steps: First we normalize our image by subtracting the background in a 145 × 145 Pixel window and rescaling the intensity values between 0.0 and 1.0 . Next - based on the methods described in [38] we calculate a local gradient $G_{x}$ and $G_{y}$ in X- and Y-direction respectively. A first orientation estimate $\phi_{FI}$ (I index for initial) can be derived via

(12) $$\phi_{FI}(x,y)=\arctan\left(\frac{2\times G_{xy}}{(G_{xx}-G_{yy})+\sqrt{{\left(G_{xx}-G_{yy}\right)}^{2}+{\left(2\times G_{xy}\right)}^{2}}}\right),$$

with

(13) $$\left[\begin{array}{c} G_{xx}(x,y)\\ G_{yy}(x,y)\\ G_{xy}(x,y) \end{array}\right]=\frac{1}{{\left(2\times N+1\right)}^{2}}\times \sum_{i=-N}^{N}\sum_{j=-N}^{N}\left[\begin{array}{c} G_{x}{\left(x+i,y+j\right)}^{2}\\ G_{y}{\left(x+i,y+j\right)}^{2}\\ G_{x}(x+i,y+j)\times G_{y}(x+i,y+j) \end{array}\right],$$

with N being the half-size of the sliding window which in our case was set to 7. Note that retinal vessel locations are excluded from this calculation in order to not incorporate the gradient direction of those into the map. As seen in Fig. 4(B) this first estimate is very noisy and in some areas contains wrong orientation estimates. Since we expect the Jansonius mathematical model to be roughly accurate, but not precise, we compare our first orientation estimate with the data from this mathematical model to get a measure of the difference $DST_{m}$ to the model via

(14) $$DST_{m}(x,y)=\sqrt{{\left(\sin\left(2\times \phi_{FI}\right)-\sin\left(2\times \phi_{M}\right)\right)}^{2}+{\left(\cos\left(2\times \phi_{FI}\right)-\cos(2\times \phi_{M})\right)}^{2}}.$$

Additionally we calculate the measure of difference $DST_{n}$ to the neighbour, checking how well the orientation at any point (x,y) fits its neighbouring points within a certain window via

(15) $$DST_{n}(x,y)=\sqrt{s_{fit}^{2}+c_{fit}^{2}},$$

with

(16) $$\left[\begin{array}{c} s_{fit}(x,y)\\ c_{fit}(x,y) \end{array}\right]=\left[\begin{array}{c} \sin{\left(2\times \phi_{FI}(x,y)\right)}^{2}\\ \cos{\left(2\times \phi_{FI}(x,y)\right)}^{2} \end{array}\right]\times {\left(\frac{1}{{\left(2\times N+1\right)}^{2}}\times \sum_{i,j=-N}^{N}\left[\begin{array}{c} \sin\left(2\times \phi_{FI}(x+i,y+j)\right)\\ \cos\left(2\times \phi_{FI}(x+i,y+j)\right) \end{array}\right]\right)}^{2},$$

with N being the half size of the Window. These two difference factors $DST_{m}$ and $DST_{n}$ are then combined and averaged to serve as weights for a dynamically sized weighted local average filter of the originally derived orientation $\phi_{FI}$ via

(17) $$W(x,y)=1-\frac{1}{2}\times \sqrt{\bar{DST_{m}}{\left(x,y\right)}^{2}+\bar{DST_{n}}{\left(x,y\right)}^{2}},$$

with $\bar{DST_{m}}$ and $\bar{DST_{n}}$ being sliding window average filtered versions of $DST_{m}$ and $DST_{n}$ respectively. Finally the actual orientation estimation $\phi_{F}$ of the RNFB projection can be obtained using a large weighted window average of the sin and cos components of the initial estimate $\phi_{FI}$ .

(18) $$\phi_{F}(x,y)=\frac{1}{2}\times \arctan\left(\frac{s_{\phi }(x,y)}{c_{\phi }(x,y)}\right),$$

with

(19) $$\left[\begin{array}{c} s_{\phi }(x,y)\\ c_{\phi }(x,y) \end{array}\right]=\sum_{i,j=-N}^{N}W(x+i,y+j)\times \left[\begin{array}{c} \sin\left(2\times \phi_{FI}(x+i,y+j\right)\\ \cos\left(2\times \phi_{FI}(x+i,y+j\right) \end{array}\right].$$

Note that the window size N is not the same for the entire image, but instead dynamically gets smaller for positions (x,y) near and at the fovea. Since at the fovea nerve fiber bundles come together from various directions, a large average window there would wash out these high orientation differences. The dynamic reduction of window size N is implemented by checking for every position (x,y) whether the window centered around it with the size N would cover the position of the fovea. If so, N is reduced for this position, such that the window does not include the fovea anymore. The final orientation map can be seen in Fig. 4(C).

### 2.5 Combining orientation data

After obtaining orientation estimation data $\phi_{M}$ , $\phi_{P}$ and $\phi_{F}$ from the mathematical model, the polarization data and the RNFB projection image respectively a global orientation map can be created. Except for $\phi_{M}$ all calculations were made on a per retinal scanning region basis - meaning that first we need to create a single large orientation map for the polarization and RNFB projection maps respectively, which is done using the methods we described in our previous paper [28]. Before the 3 large FOV orientation maps can be combined, however, the axis offset ofs of $\phi_{P}$ , as mentioned in section 2.4.2 has to be corrected. For this we take a broad circular band around the ONH and compare $\phi_{F}$ with $\phi_{P}+ofs$ . To be precise we calculate the sum of squared differences of the double sin⁡() and cos⁡() component of both orientation maps - offsetting $\phi_{P}$ by an angle from -90° to +90° in increments of 1. The offset ( ofs ) that yields the lowest squared difference will then be applied to $\phi_{P}$ .

Finally we can join the two large field maps $\phi_{F}$ and $\phi_{P}$ into the orientation map OM . To determine the contribution of the two maps for a given position (x,y) we once again compare them to the mathematical model, using

(20) $$W(x,y)=1-\frac{1}{\sqrt{2}}\sqrt{{\left(sin\left(2\times \phi \right)-sin\left(2\times \phi_{M}\right)\right)}^{2}+{\left(cos\left(2\times \phi \right)-cos\left(2\times \phi_{M}\right)\right)}^{2}},$$

which is calculated once for $\phi =\phi_{P}$ giving us $W=W_{P}$ and once for $\phi =\phi_{F}$ giving $W=W_{F}$ . After smoothing the weight contributions $W_{P}$ and $W_{F}$ with a sliding average filter, they are joined via

(21) $$OM(x,y)=\frac{1}{2}\times \arctan\left(\frac{s_{joined}}{c_{joined}}\right)$$

with

(22) $$\left[\begin{array}{c} s_{joined}(x,y)\\ c_{joined}(x,y) \end{array}\right]=\frac{1}{W_{p}+W_{f}}\times \left[\begin{array}{c} W_{p}\times \sin\left(2\times \phi_{P}\right)+W_{f}\times \sin\left(2\times \phi_{F}\right)\\ W_{p}\times \cos\left(2\times \phi_{P}\right)+W_{f}\times \cos\left(2\times \phi_{F}\right) \end{array}\right].$$

### 2.6 RNFB tracing

Given the combined large field orientation map OM(x,y) the RNFB trajectories can be traced. Trajectories are calculated based on line integration by taking the angle at a position (x,y) and moving a set amount in that direction, resulting in the new position $\left(x^{\prime },y^{\prime }\right)$ from which point the process can be repeated until a certain termination point is reached. The equation for this is simply

(23) $$\left[\begin{array}{c} x^{\prime }\\ y^{\prime } \end{array}\right]=r\times \left[\begin{array}{cc} \cos\left(OM(x,y)\right) & -\cos\left(OM(x,y)\right)\\ \sin\left(OM(x,y)\right) & -\sin\left(OM(x,y)\right) \end{array}\right],$$

with r being the step size in pixel. Note that we calculate 2 potential positions for $\left(x^{\prime },y^{\prime }\right)$ since we cannot know whether e.g. an angle of 0° points left or right. The problem of choosing the correct direction is linked to the problem of choosing the best starting point. Intuitively one might want to trace RNFBs starting from a set of positions located in a circle around the ONH in which case we would always choose the $\left(x^{\prime },y^{\prime }\right)$ that is further away from the center of the ONH. Since the path of RNFBs outwards of the ONH is divergent, this means that small inaccuracies of our orientation map will also diverge - thus increasing the error over the course of a trace. The more robust method is to trace inwards - meaning from the boundaries towards the ONH in which case we will always choose the $\left(x^{\prime },y^{\prime }\right)$ with the minimum distance to the center of the ONH.

In practice, however, the most probable use-case is that a specific position of interest (e.g. a visual field test coordinate) is selected in the image and the trace passing through that point has to be determined. In this case we will simply calculate 2 sub-traces - One following the trajectory towards the ONH and one away from it - and then combine them.

## 3. Results

**Fig. 5.**
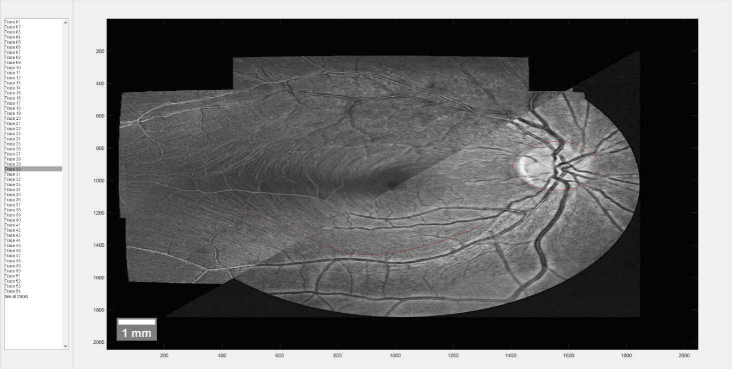
Custom tool for manual RNFB tracing. Individual visual field coordinates can be selected on the left, sample trace is drawn in in red. Top left shows intensity variation projection, bottom right shows retinal fundus image. Between the two can be toggled at any time.

The accuracy of our RNFB tracing method was evaluated as follows. A subset of 7 out of 123 healthy subjects was selected for manual RNFB tracing which was performed by 3 trained clinical experts as manual graders using a custom-built manual tracing tool. Similar to the evaluation in [44] we matched the coordinates of the 24-2 humphrey visual field test to the retinal fundus image (using translation, rotation and scaling) such that the fovea and ONH-center locations of the two were superimposed. The manual graders were shown one such visual field coordinate at a time and were tasked with tracing the entire path up to the ONH for each visual field point. Graders were free to zoom in and toggle between a retinal fundus image or the stitched projection image of RNFL intensity near the RNFL/GCL boundary at any time during the process (Fig. 5).

In order to get a measure of how well the different graders agree in their respective tracings, we perform an analysis similar to the one in [44]. For this we calculate the entry angle (EA) to the ONH (which is defined as the angle at which the trace enters an area within a radius of 4° around the center of the ONH) and then compare these EA between graders. We calculate an EA range of the 3 graders (which is simply the maximum difference between any two graders defined as max(EA)−min(EA) , compensated for the 360° wraparound), as well as an average EA of the 3 graders for any trace to get the offset between the EA of every grader and the average EA. In addition, we calculate the intra-class correlation coefficient (ICC) of the EA for the 3 graders using the "A-k" scheme (again compensated for the 360° wraparound) to confirm the reliability of the obtained results. Furthermore we want to analyze the variability between graders along the entire trace. We first transformed all traces to a polar coordinate system centered around the center of the ONH and interpolated the trace points using linear interpolation. Next an average trace for all 3 graders was calculated by averaging the 3 interpolated angles for every radius at fixed intervals. Given an averaged trace $T_{A}$ we can calculate the root mean square error (RMSE) between it and the trace of any grader T(G) (with G∈N (1,3) ). To do this we take every radial position r of the traces, denoted as $T_{A}(r)$ and T(G,r) for the averaged trace and the each of the graders’ traces respectively and calculating the squared distance between each graders’ trace and the average trace to get an overall RMSE via:

(24) $$RMSE=\frac{1}{3}\times \sum_{G=1}^{3}\sqrt{\frac{1}{length(T_{A})}\times \sum_{r=4}^{length(T_{A})}\left(\min\left({\left(T_{A}\left(r\right)-T\left(G,r\right)\right)}^{2}\right)\right)}.$$

For analysis of the results we collected the traces into 6 groups depending on their individual VF starting points based on the Garway-Heath scheme [45]. Table 1 shows the detailed results of that analysis.

**Table 1.** Expert grader comparison of EA and trace overlap including standard deviation (SD), separated into sectors temporal (T), superior temporal (ST), inferior temporal (IT), superior nasal (SN), inferior nasal (IN), nasal (N) and overall

Sector	EA avg range	EA avg offset	trace RMSE	EA ICC
T	11.6° (SD=8.6)	4.5° (SD=3.4)	78 μm (SD=63)	0.997
ST	23.6° (SD=12.1)	9.1° (SD=4.8)	141 μm (SD=70)	0.910
IT	16.4° (SD=11.6)	6.4° (SD=4.6)	141 μm (SD=78)	0.915
SN	13.6° (SD=9.8)	5.3° (SD=3.8)	92 μm (SD=67)	0.828
IN	15.5° (SD=9.8)	6.0° (SD=3.7)	117 μm (SD=71)	0.858
N	7.9° (SD=4.3)	3.1° (SD=1.6)	45 μm (SD=23)	0.957
Overall	16.0° (SD=11.4)	6.2° (SD=4.4)	112 μm (SD=75)	0.989

Using the graders’ traces as ground truth we can assess the accuracy of our automatic method, as well as our implementation of the mathematical model by Jansonius et. al. . For this we take the same visual field coordinates as we used for manual tracing and performed automatic tracing in direction of the ONH once using the mathematical model and once using our proposed method. RMSE is calculated the same way as previously, by comparing each point along the trace T(r) to the corresponding point along the averaged grader trace $T_{A}(r)$ , except that we don’t need to average this over multiple graders. Thus Eq. (24) can be modified into

(25) $$RMSE=\sqrt{\frac{1}{length(T_{A})}\times \sum_{r=4}^{length(T_{A})}\left(\min\left({\left(T_{A}\left(r\right)-T\left(r\right)\right)}^{2}\right)\right)},$$

**Fig. 6.**
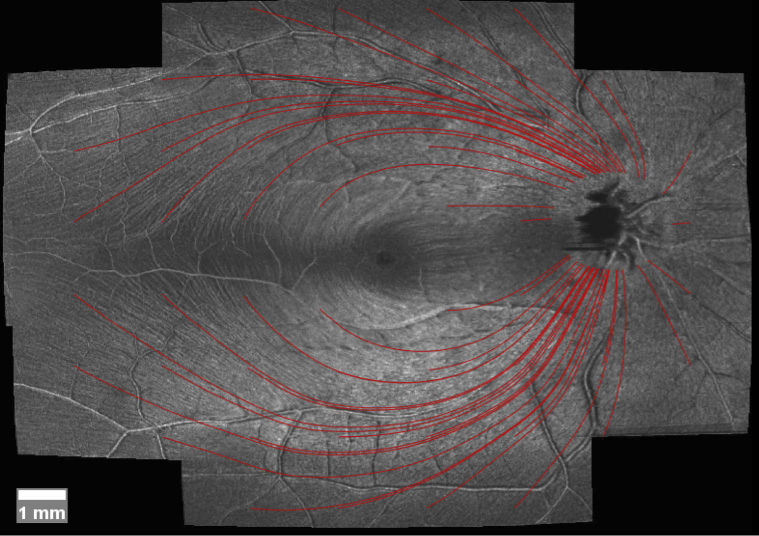
Projection of the RNFL, averaged near the RNFL/GCL boundary. Tracings derived via our proposed method starting from 24-2 Humphrey VF points drawn in.

Table 2 shows the results of this analysis, again separated into groups. The complete automatic tracing using our proposed method can be seen in Fig. 6 for a healthy subject.

**Table 2.** Comparison of automatic tracing with the mathematical model

	our method		mathematical model	
Sector	EA avg offset	trace RMSE	EA avg offset	trace RMSE
T	6.1° (SD=5.0)	97 μm (SD=83)	4.6° (SD=4.0)	108 μm (SD=121)
ST	10.1° (SD=8.7)	185 μm (SD=123)	9.0° (SD=7.0)	229 μm (SD=163)
IT	10.0° (SD=7.6)	241 μm (SD=129)	6.4° (SD=5.0)	277 μm (SD=171)
SN	9.2° (SD=8.1)	243 μm (SD=191)	17.2° (SD=27.1)	228 μm (SD=239)
IN	8.9° (SD=6.0)	202 μm (SD=93)	5.6° (SD=3.5)	214 μm (SD=155)
N	3.2° (SD=3.0)	43 μm (SD=33)	21.1° (SD=35.0)	75 μm (SD=61)
Overall	8.6° (SD=7.5)	188 μm (SD=145)	9.8° (SD=16.6)	210 μm (SD=183)

Finally, we can test the reproducibility of our automatic tracing method. For a subset in our study consisting of 5 healthy subjects and 6 glaucoma patients, we have acquired 3 complete sets of wide field OCT data on different days (average time difference in the sample was 19 days). These datasets are processed completely independent of one another, except for the retinal fundus image, which is the same for all 3 sets and is used for our registration and stitching [28]. The question of reproducibility of one algorithm in 3 different datasets of the same eye is actually the same as the question of variability of 3 different manual graders on the same dataset of one eye. Thus we used the exact same methods used for the analysis in Table 1 for this test, which are presented in Table 3.

**Fig. 7.**
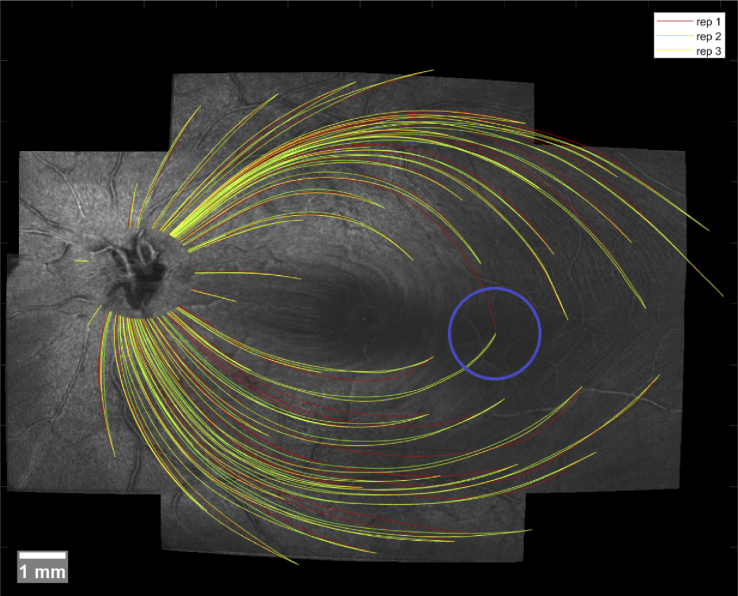
Automatic traces of repeated scans of a subject. One VF starting point was mistraced in the wrong direction (inside blue circle).

In the case of the repeated measurements of Table 3 there was a total of 3 cases (1 healthy, 2 glaucoma) where one VF starting point was located so close to the raphe such that it was ambiguous whether to start tracing upwards or downwards (as seen in Fig. 7). In all 3 cases this VF point was located in the "IT" group and it caused one repetition to trace in the opposite direction to the other 2 repetitions, thus causing a severe increase in trace RMSE, as well as EA range and offset. We decided to repeat these calculations with the affected 3 traces excluded to get a clearer understanding of the algorithm performance. Table 4 shows the results after excluding the traces for "IT" and "Overall" - all other regions remain unaffected by this change.

**Table 3.** Repeatability comparison of the automatic tracing with the averaged manual trace

	Healthy subjects			
Sector	EA avg range	EA avg offset	trace RMSE	EA ICC
T	4.7° (SD=3.5)	1.8° (SD=1.4)	33 μm (SD=30)	0.999
ST	5.1° (SD=3.7)	1.9° (SD=1.3)	54 μm (SD=31)	0.996
IT	8.9° (SD=12.2)	3.4° (SD=5.3)	106 μm (SD=202)	0.944
SN	5.5° (SD=4.6)	2.0° (SD=1.6)	56 μm (SD=32)	0.987
IN	6.5° (SD=3.0)	2.5° (SD=1.0)	67 μm (SD=42)	0.982
N	3.6° (SD=3.1)	1.3° (SD=1.1)	19 μm (SD=15)	0.999
Overall	6.1° (SD=7.0)	2.3° (SD=2.9)	63 μm (SD=106)	0.996

Glaucoma patients				
	
T	6.0° (SD=4.8)	2.3° (SD=1.8)	42 μm (SD=36)	0.999
ST	5.7° (SD=3.6)	2.2° (SD=1.4)	48 μm (SD=27)	0.996
IT	13.7° (SD=21.3)	5.3° (SD=8.8)	141 μm (SD=290)	0.938
SN	5.5° (SD=3.2)	2.1° (SD=1.2)	52 μm (SD=28)	0.990
IN	11.0° (SD=6.7)	4.3° (SD=2.7)	78 μm (SD=42)	0.947
N	4.6° (SD=3.0)	1.8° (SD=1.2)	24 μm (SD=16)	0.999
Overall	8.4° (SD=11.8)	3.2° (SD=4.8)	73 μm (SD=152)	0.994

**Table 4.** Repeatability comparison after faulty VF trace exclusion

Healthy subjects				
	
Sector	EA avg range	EA avg offset	trace RMSE	EA ICC
IT	7.4° (SD=4.0)	2.8° (SD=1.5)	82 μm (SD=53)	0.998
Overall	5.8° (SD=4.0)	2.2° (SD=1.4)	57 μm (SD=42)	0.999

Glaucoma patients				
	
IT	10.5° (SD=8.9)	4.0° (SD=3.1)	95 μm (SD=62)	0.993
Overall	7.6° (SD=6.1)	2.9° (SD=2.4)	62 μm (SD=47)	0.999

## 4. Discussion

We presented a new method for fully automatically tracing the trajectory of retinal nerve fiber bundles on stitched large FOV PS-OCT data. The algorithm itself works on any kind and size of PS-OCT volume, accurate stitching, however, allows even longer nerve fiber bundle trajectories to be traced.

Our orientation maps are generated using polarization data and intensity data of the RNFL, as well as a mathematical model to improve stability. Given that we need to access information from specific retinal layers it is of importance to provide an accurate and stable retinal layer segmentation which can prove difficult in low SNR data. The method of visualizing nerve fiber bundles using intensity data is dependent on the nerve fiber bundles having a significantly higher average intensity than the background. This effect will be greatly reduced with lower SNR and in the case of glaucoma [46], increasing the difficulty in extracting information.

A major point of weakness in our proposed method could be the use of fingerprint orientation estimation techniques. As discussed earlier, our intensity variation projection images bear similar features to fingerprint images which encouraged us to use these methods. One difference to the fingerprint case is the presence of additional different structural information in form of retinal vessels. For orientation estimation they are of course segmented and excluded, this process unfortunately, is not perfect and sometimes leaves false gradient information that can negatively influence the estimation process. The bigger difference, however, is the lack of uniformity in the spacing between RNFBs compared to fingerprint ridges. In typical fingerprint images the spacing between ridges and valleys remains pretty much constant over the entire image, whereas RNFBs tend to spread outwards the further they are from the ONH. This means that near the ONH they are squeezed so tightly, that neighboring bundles may occupy the same pixel on the image making it difficult to extract a gradient between them. It also means that further out - and this was especially noticed near the raphe - they are spread out very far, leaving large areas in between bundles with no usable gradient information due to the lack of bundles there which causes problems when creating an averaged orientation map. Another problem is the already mentioned lowered SNR of the OCT volume in cases of glaucoma, which negatively affects the reflectivity of the RNFBs, making it harder to extract orientation information using regular fingerprint orientation estimation techniques. Thus other methods, or additional modifications to the fingerprint orientation estimation techniques may need to be implemented to improve the accuracy and robustness of our method.

In our work we use the mathematical model as proposed by Jansonius et. al. to fill in the gaps in the estimated RNFB orientation map. If for a certain retinal region neither the axis orientation data from the PS-OCT, nor the orientation estimation from the intensity variation projection is usable, we blend in the mathematical model via interpolation. This is also true for regions where a RNFB trajectory briefly goes out of bounds of our stitched image (mainly the inner corners of our "cross" form). When generating our fingerprint-based orientation map we also rely on the mathematical model. This may introduce a slight bias or trend of our generated orientation map towards orientation map based on the mathematical model. Judging from our results, however, it is clear that our method outperforms the pure mathematical model. The mathematical model is presently still based on manual tracing and consequently inherently less accurate compared to our automatic tracing as our data show. For this reason it is expected that in more severe glaucoma cases, where a larger part of the tracing has to rely on the mathematical model this will have an effect on the results. However for the future we plan to redesign the mathematical model based on automatic traces in a larger number of healthy subjects and this redesign will also include a personalization, based on confounders like the positions of the major retinal blood vessels and axial eye length. Also the mathematical model is based on healthy eyes only and it cannot be excluded that the trajectories might be different in glaucoma as compared to healthy eyes although this seems rather unlikely to us.

The 3 trained graders were very consistent in their tracing results, only deviating on average 6.2° from their respective average entry angle of the ONH as can be seen in Table 1. The worst concordance was achieved in the ST and IT Garway-Heath sectors with 9.1° and 6.4° respectively, which is not surprising, considering that these two sectors cover the RNFBs with the longest pathways in our FOV. Additionally, the dominant retinal vessel arch reportedly increased the tracing difficulty significantly. The same pattern can also be seen when analyzing the RMSE along the traces where again the ST and IT sectors are above the average of 112 μm with 141 μm each. Interestingly, the EA ICC is still quite high even in those regions with values of 0.910 and 0.915 respectively and instead gets lower for SN and IN with only 0.828 and 0.858 respectively. Overall, however, the EA ICC also shows a high consistency of the graders’ tracing results with a value of 0.989.

Comparing the mathematical model and our method to the reference results of the manual grading it can be seen that the average EA offset overall is with 8.6° slightly lower than of the mathematical model with 9.8°, the SD of those offsets, however, is less than half with 7.5° to 16.6° respectively indicating considerably less variation in between subjects. Similarly the RMSE along the trace is quite a bit lower overall than of the mathematical model with 188 μm (SD=145) versus 210 μm (SD=183) respectively, as presented in Table 2.

When testing for repeatability, Tables 3 and 4 show that the EA avg offset is almost reduced to a third for healthy subjects with 2.2° and more than halved for glaucoma patients with 2.9° compared to manual grading with 6.2°. The EA ICC as well shows higher concordance of the repeated measurements than the manual grading with an overall value of 0.999 for healthy subjects as well as glaucoma patients. One has to keep in mind, however, that manual grading was only performed on healthy subjects, so the repeatability on glaucoma patients cannot directly be compared. We can see that the EA offset, as well as the trace RMSE is slightly higher on the glaucoma set than on the healthy set, which is to be expected, since patient scans often tend to have a lower signal quality than scans of healthy subjects.

As mentioned earlier there was a total of 3 repeated traces that had to be excluded because they run in opposite directions, as shown in Fig. 7. This only affected starting points very close to the raphe where the true trajectory can sometimes be ambiguous. This problem may be reduced by improving the orientation estimation of the intensity variation projection images as discussed previously. It should be mentioned, however, that even the clinical expert graders on initial training data inspection during training did not agree whether the trajectory goes up or down on some starting points close to the raphe. This just shows that the raphe can be a particularly difficult region for tracing.

We acknowledge the mentioned limitations to our study work, as well as our very limited set of data. This applies to both - our limited set of manually graded subjects, as well as the set of repeated measurements. As a proof of concept, however, we show that stitched large FOV PS-OCT data can be used for fully automatic individualized RNFB tracing on both healthy subjects and glaucoma patients. More work has to be done especially in fundus areas near the raphe and the presence of large vessels to further improve the method.

## 5. Conclusion

PS-OCT provides additional information on RNFB structures, giving the possibility to reconstruct trajectories of RNFBs more accurately. In contrast to mathematical models or even manual grading, we integrate the information from 3 different sources to construct the individual traces from measurements. Our preliminary results state that our method is superior to the mathematical model which is limited in individuum specific parametrization. Furthermore the results of our method are free from grader variability and highly reproducible. This will also open the way to analyse clinical parameters along the trajectory of arbitrary individual RNFBs without the time consuming manual grading.

## Data Availability

Data underlying the results presented in this paper are not publicly available at this time, but may be obtained from the authors upon reasonable request.
